# Reversible Rapidly Progressive Dementia Due to Cerebral Amyloid Angiopathy

**DOI:** 10.7759/cureus.110017

**Published:** 2026-06-01

**Authors:** Godknows Osarhiaekhimen, Ayomide Ayo-Salami, Adeolu Morawo

**Affiliations:** 1 Internal Medicine, Tertiary Education Trust Fund, Abuja, NGA; 2 Neurology and Internal Medicine, American University of Integrative Sciences, Atlanta, USA; 3 Neurology, CHI Health St. Francis, Grand Island, USA

**Keywords:** autoimmune encephalitis, caa-related inflammation( caa-ri), cerebral amyloid angiopathy (caa), inflammatory infiltrates, rapidly progressive dementia

## Abstract

Cerebral amyloid angiopathy (CAA) is a vascular disorder of the brain caused by beta-amyloid deposits in small- and medium-sized vessels. In some patients, the condition triggers an inflammatory response, which can appear in different forms based on the pattern of vessel involvement. These rare but treatable inflammatory variants may present with a syndrome of rapidly progressive dementia, which may pose a diagnostic challenge. Here, we describe a 76-year-old man who experienced two distinct episodes of rapidly progressive cognitive deterioration accompanied by diffuse weakness and seizure activity, separated by a seven-year interval. Despite extensive diagnostic evaluation during the initial presentation, a definitive diagnosis was not established. During the second episode, advanced neuroimaging revealed characteristic asymmetric white matter changes, and subsequent histopathologic examination confirmed cerebral amyloid angiopathy-related inflammation (CAA-ri). This case underscores the diagnostic complexity of CAA-ri and highlights the importance of clinicoradiologic correlation and tissue confirmation in atypical or recurrent presentations of rapidly progressive dementia.

## Introduction

Cerebral amyloid angiopathy (CAA) is a frequent age-associated cerebrovascular disorder characterized by amyloid-beta deposition in the walls of small- to medium-sized cortical and leptomeningeal vessels. While it is most commonly linked to cognitive decline and lobar intracerebral hemorrhage in the elderly, a subset of patients develops an inflammatory variant with a distinct clinical and pathologic profile. Inflammatory CAA is categorized into two subtypes: cerebral amyloid angiopathy-related inflammation (CAA-ri), defined by perivascular lymphocytic infiltration, and amyloid-beta-related angiitis (ABRA), which features granulomatous inflammation involving the full thickness of the vessel wall. These subtypes are indistinguishable clinically and radiologically and can only be differentiated based on histopathology. They also have similar prognoses. It is hypothesized that they exist on a spectrum of central nervous system (CNS) vasculopathies from CAA to primary angiitis of the CNS [1]. CAA-ri is rare but potentially life-threatening. It arises from β-amyloid accumulation in the media and adventitia of affected vessels, provoking an immune-mediated inflammatory response that leads to vascular injury and impaired cerebral perfusion. This structural compromise increases the risk of cortical-subcortical or lobar hemorrhages, which are diagnostic markers of inflammatory CAA and are associated with a high risk of recurrence [2]. Clinically, CAA-ri often presents with rapidly progressive cognitive decline, seizures, focal neurologic deficits, and headaches, mimicking other encephalopathies. Additionally, remission could occur without appropriate treatment, and the clinical presentation could follow a relapsing course. These factors acting in concert contribute to the challenge of diagnosing this entity. Early recognition is crucial to initiating appropriate therapy and improving patient outcomes. Although biopsy provides conclusive evidence of CAA-ri, key diagnostic clues, including distinct radiologic patterns together with clinical presentation, are central to raising suspicion for possible CAA-ri, which is supported by a good response to immunosuppressive treatment. A poor response to immunosuppressive treatment after a probable CAA-ri diagnosis warrants a brain biopsy [1,2].

This case report seeks to emphasize the distinguishing clinical and radiologic features of CAA-ri, highlight the reversibility of this clinical syndrome without appropriate treatment, the possibility of an unusually long interval before recurrence, and the importance of a comprehensive magnetic resonance imaging (MRI) protocol in the workup, enabling its clear-cut differentiation from other forms of rapidly progressive dementia.

This case was initially presented at the American Academy of Neurology 2025 Annual Meeting as a poster.

## Case presentation

A 76-year-old male presented to our facility with rapidly progressive cognitive impairment, generalized weakness, and a seizure episode in 2024. Seven years prior (2017), he had presented similarly with rapidly progressive cognitive decline and other symptoms, which included new-onset headache, hallucinations, bizarre thoughts, compulsive fixation on weight-bearing surfaces with his hands, an inability to multitask, and weight loss evolving over weeks, to an outside hospital. MRI of the brain performed at that time did not include susceptibility-weighted (blood-sensitive) sequences and was otherwise unremarkable. The patient had undergone blood and cerebrospinal fluid (CSF) testing, including for autoimmune encephalitis, infectious encephalitis, toxic-metabolic encephalopathy, and prion disease, which were unrevealing except for a weakly positive channel-binding antibody in CSF, which was deemed at that time non-diagnostic (Table 1).

**Table 1:** Serum and cerebrospinal fluid evaluation done on initial presentation in 2017 *Outside the reference range WBC, white blood cells; RTQuic, real-time quaking-induced conversion

Body fluid	Parameter	Value	Reference range
Serum	Calcium channel-binding antibody*	0.05 nmol/L	<0.03 nmol/L
	C-reactive protein*	2.5 mg/dL	<0.3 mg/dL
	Erythrocyte sedimentation rate*	53 mm/hr	40 mm/hr (for older adults)
	Anti-nuclear antibody	1:40	1:40 or lower
Cerebrospinal fluid	WBC*	10/uL	0-5 cells/uL
	Lymphocytes*	94%	40-80%
	Monocytes*	6%	15-45%
	14-3-3 protein	Negative	Negative
	RTQuic	Negative	Negative

The patient was eventually diagnosed with autoimmune encephalitis of unknown origin and, with progressively worsening cognitive decline, was subsequently discharged to hospice, but he soon spontaneously recovered, returned to his premorbid baseline, and resumed normal life.

However, in 2024, seven years after the first episode, he re-presented to our facility with a similar clinical syndrome. MRI of the brain performed on admission, including fluid-attenuated inversion recovery (FLAIR) and gradient-recalled echo (GRE) sequences, demonstrated asymmetric cortical-subcortical hyperintensities involving the right cerebral hemisphere with associated sulcal effacement, consistent with vasogenic edema (Figure 1A). GRE sequences revealed multiple cortical and subcortical hypointense foci, in keeping with cerebral microbleeds (CMBs) (Figure 1B). A brain biopsy confirmed the presence of CAA-ri (Figure 2).

**Figure 1:**
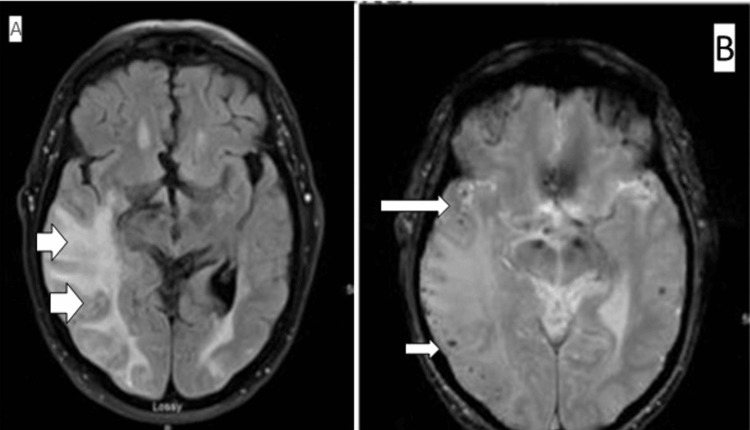
MRI with FLAIR and GRE sequences obtained on admission in 2024 (A) Axial image with FLAIR reveals asymmetric cortical-subcortical hyperintensities involving the temporal and frontal lobes of the right cerebral hemisphere with associated sulcal effacement, consistent with asymmetric vasogenic edema. (B) Axial image with GRE showing multiple cortical and subcortical hypointense foci involving the right temporal and frontal lobes, consistent with cerebral microbleeds. FLAIR, fluid-attenuated inversion recovery; GRE, gradient-recalled echo

**Figure 2:**
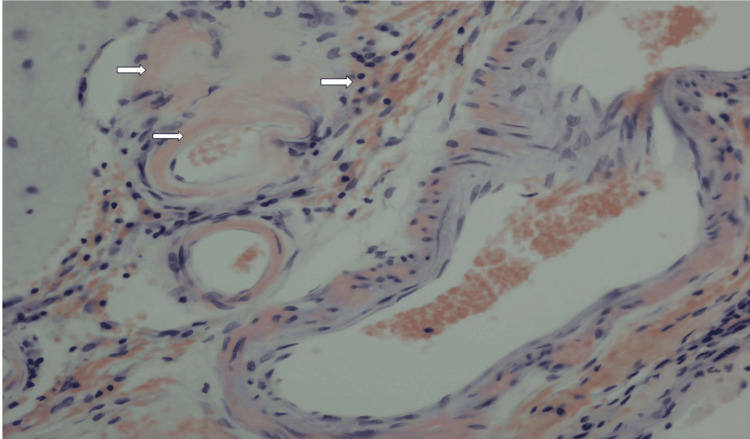
Histopathologic examination of a brain biopsy obtained at index presentation in 2024 Brain biopsy demonstrates polarizable amorphous deposits positive on Congo red special stain, affecting small blood vessels, compatible with vascular amyloid deposition. These deposits are more marked in the intraparenchymal small blood vessels, although leptomeningeal small blood vessels are also affected. Perivascular hemosiderin deposition compatible with blood vessel damage is present, along with associated perivascular lymphocytic inflammation and giant cells. Total destruction of the blood vessel wall is not identified.

Histopathologic examination of a brain biopsy obtained at index presentation demonstrated thickened small vessel walls containing eosinophilic material, suggestive of amyloid deposition, with associated perivascular inflammatory infiltrates, supporting the diagnosis of CAA-ri (Figure 2).

Treatment consisted of high-dose intravenous methylprednisolone for five days, followed by an oral steroid taper. Six weeks after the initiation of steroid therapy, the patient was back to his cognitive baseline. Follow-up MRI of the brain obtained six weeks after initiation of corticosteroid therapy demonstrated near-complete resolution of the previously observed asymmetric cortical-subcortical FLAIR hyperintensities and vasogenic edema (Figure 3A) [3]. Persistent cortical and subcortical susceptibility foci on GRE sequences were again noted, consistent with underlying CMBs characteristic of CAA (Figure 3B).

**Figure 3:**
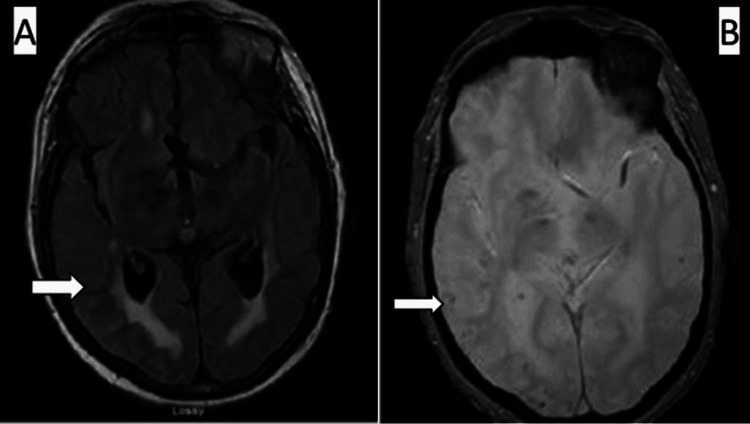
Follow-up brain MRI with FLAIR and GRE obtained six weeks after initiation of steroid therapy (A) Axial FLAIR sequence reveals interval near-resolution of the previously noted asymmetric cortical-subcortical hyperintensities and vasogenic edema. (B) Axial GRE sequence demonstrates multiple persistent susceptibility foci, consistent with cerebral microbleeds characteristic of CAA. CAA, cerebral amyloid angiopathy; FLAIR, fluid-attenuated inversion recovery; GRE, gradient-recalled echo

## Discussion

CAA refers to a broad category of cerebrovascular pathologies characterized by the deposition of beta-amyloid proteins in the walls of small blood vessels supplying the cerebral cortex and leptomeninges [4]. CAA could be hereditary, as seen in patients with Down syndrome, or sporadic, where it is age-related, as in this case.

Although 10%-40% of elderly patients are found to have beta-amyloid deposits in leptomeningeal and cortical small vessels, sporadic CAA-ri is a relatively rare clinical entity, with the average age of onset falling within the seventh decade of life. This is in line with the initial presentation of our patient. There is no obvious gender predilection [1,4,5]. The prevalence was 0.13/100,000 in a Japanese survey [6].

CAA can be classified into inflammatory and non-inflammatory types. The inflammatory type, associated with vascular inflammation, can be subclassified into inflammatory CAA (ICAA)/CAA-ri and ABRA. Although these subtypes have distinct histopathologic findings, they can be seen as being on a spectrum progressing from CAA to primary angiitis of the central nervous system (PACNS) due to the overlap in their radiologic findings and clinical manifestations [4].

CAA is twice as likely to manifest with intracerebral hemorrhages as ischemic strokes [7]. The clinical features of CAA-ri often present for weeks to months before diagnosis [1]. A systematic review carried out in 2015 found that over 60% of patients presented clinically with symptoms that had lasted over a month [8]. The most common symptom is a subacute decline in cognitive function, which was notably the primary symptom in this case. Other symptoms include seizures ranging from focal seizures to status epilepticus, headache, and encephalopathy. Focal neurologic deficits could result from microbleeds and frequently present with visual symptoms, hemiparesis, aphasia, and ataxia [4,7]. Our patient presented with diffuse weakness and a seizure. Parkinsonism has been reported in an elderly patient with CAA-ri and was thought to be caused by subcortical lacunar infarcts resulting from vascular occlusion [9].

CAA-ri is hypothesized to result from an autoimmune reaction to beta-amyloid deposition in the media and adventitia of cortical and leptomeningeal blood vessels. High titers of antibodies against beta-amyloid have been found in the blood and CSF of patients with inflammatory CAA and have been found to decline after steroid therapy, alluding to their role in mediating this encephalopathy and a possible future role in making a diagnosis and monitoring response to therapy. On histopathologic analysis of brain and leptomeningeal tissue, the characteristic findings of vascular amyloid deposits surrounded by an inflammatory infiltrate consisting of T lymphocytes and microglia also lend credence to the autoimmune response hypothesis [1,4,7]. This inflammatory response results in loss of integrity of the vessel walls, leading to vessel wall rupture and culminating in CMBs, hematoma formation, or vascular occlusion [5].

The ApoE ε4/ε4 homozygous genotype, a known genetic risk factor for sporadic Alzheimer's disease, is found in 75% of patients with CAA-ri, suggesting a pro-inflammatory effect in addition to its beta-amyloid deposition-promoting effect. ARIA is also more likely to be seen in ApoE ε4 carriers following anti-beta-amyloid treatment [1]. Although ApoE ε4 homozygosity is strongly associated with CAA-ri and may increase diagnostic confidence, it has no standalone diagnostic utility [10]. In this patient, who met clinicoradiologic criteria for probable CAA-ri with histopathologic confirmation, it would not have added anything new to management and monitoring. Elevated CSF anti-β-amyloid autoantibodies provide supportive evidence for CAA-ri and reinforce its proposed autoimmune pathogenesis; however, these biomarkers remain investigational and are not currently included in validated diagnostic criteria [7].

Definitive diagnosis of CAA-ri remains reliant on histopathologic confirmation through brain biopsy, which is considered the gold standard. Tissue examination typically reveals vascular amyloid-beta deposits similar to those seen in sporadic CAA. A confirmed diagnosis additionally requires identification of peri- or transmural inflammatory infiltrates surrounding more than one amyloid-positive vessel. These infiltrates are composed predominantly of CD68-positive microglia and T lymphocytes [7].

Although a brain biopsy is the gold standard for a definitive diagnosis, this can be cumbersome and thus delay diagnosis and urgent treatment. Hence, neuroimaging is crucial to enable a noninvasive diagnosis of this rare encephalopathy. A set of clinical and radiologic criteria was validated in a group of biopsy-proven CAA-ri patients by Auriel et al. in 2016 and can be used to make a diagnosis of “probable CAA-ri” and “possible CAA-ri” with high sensitivity and specificity [11]. While the presence of multiple lobar CMBs supports a diagnosis of probable CAA based on Boston Criteria v2.0, the additional finding of asymmetric vasogenic edema on FLAIR imaging is more characteristic of CAA-ri. It should be noted that susceptibility-weighted imaging (SWI) is more sensitive than GRE sequencing in demonstrating superficial and deep CMBs as hypointensities. If there is a high burden of CMBs, it can be identified on T2/FLAIR. Cortical superficial siderosis may also be picked up on SWI in some cases [1,4,9]. Our case had a brain MRI performed during his second presentation that revealed asymmetric vasogenic edema in the right temporal and frontal lobes and CMBs on GRE sequencing, and the diagnosis was confirmed with a brain biopsy before initiation of therapy.

In patients with Alzheimer's disease treated with humanized monoclonal antibodies against beta-amyloid, imaging findings similar to those seen in CAA-ri have been identified as amyloid-related imaging abnormalities (ARIA). These findings include confluent vasogenic edema on FLAIR imaging, CMBs, and cortical superficial siderosis on T2-weighted GRE and SWI sequences. However, patients are asymptomatic, and CSF findings are normal [1,4].

Differential diagnosis for rapidly progressive cognitive impairment includes Creutzfeldt-Jakob disease, vascular dementia, and autoimmune encephalitis. Autoimmune encephalitis has a broader range of clinical symptomatology than CAA-ri, which includes psychiatric and movement abnormalities. Although a significant proportion of patients with autoimmune encephalitis will have negative MRI findings, hyperintensities may be seen in the limbic system on T2/FLAIR sequences. Less commonly, these hyperintensities could be seen in the cortex, brainstem, or cerebellum [12]. In this patient, the CSF returned weakly positive for calcium channel-binding antibody, suggesting autoimmune encephalitis. While this entity is a consideration in the differential diagnosis, it is uncommon for autoimmune encephalitis to resolve spontaneously without treatment [13]. Creutzfeldt-Jakob disease tends to present with myoclonus, poor coordination, movement abnormalities, and rapid cognitive decline. Hyperintensities will usually be seen in the cortex, putamen, and caudate nucleus on T2-weighted MRI, FLAIR, and diffusion-weighted imaging (DWI) sequences. Diffusion will be restricted in these affected areas on DWI sequencing [14]. In vascular dementia, periventricular and deep white matter hyperintensities will be seen on T2-weighted and FLAIR imaging, in association with cortical and subcortical infarcts.

Early diagnosis is crucial to avoid therapeutic delay. Treatment should be initiated promptly to improve clinical and radiologic outcomes and reduce the risk of recurrence. Immunosuppressants are the mainstay of therapy, and a short course of high-dose steroids, usually IV methylprednisolone, followed by a prolonged course of oral steroids for weeks to months, has been found to have favorable outcomes [1]. Relapse, defined by cognitive decline or encephalopathy after an initial improvement, may occur after a decrease in steroid dose or abrupt withdrawal of steroids. 20-30% of patients treated with the typical steroid regimen do not respond optimally [12]. Other immunosuppressants such as cyclophosphamide, azathioprine, and mycophenolate mofetil may be considered in patients showing no response to corticosteroids [4]. Over 75% of patients treated with immunosuppressants will show a positive response [15]. A retrospective cohort study carried out among CAA-ri patients found a 26% recurrence rate in patients treated with immunosuppressants compared to a 71% recurrence rate in patients who were not treated, alluding to the key role of immunosuppressants in improving prognosis and the likelihood of spontaneous recovery without treatment. However, the median time to recurrence following an untreated episode of CAA-ri was 5.2 months [10]. This is markedly different from our patient, who had a seven-year period of remission without treatment. Our patient was treated with high-dose IV methylprednisolone for five days, followed by an oral steroid taper. Follow-up MRI of the brain after treatment showed near-complete resolution of vasogenic edema, with persistence of CMBs reflecting underlying CAA.

Limitations

This case has several limitations. Notably, the initial presentation of this patient to an outside facility makes it difficult to draw certain conclusions about the workup, diagnosis, and recovery timeline of the first episode; the case presentation included only records that could be accessed regarding the clinical course of the first episode. First, the normal FLAIR findings and the absence of SWI during the initial presentation limit retrospective confirmation of CMBs and reduce diagnostic certainty regarding early CAA or CAA-ri. Second, the lack of a brain biopsy during the first episode prevents a definitive comparison between the two clinical events. Third, the weakly positive calcium channel-binding antibody introduces a potential alternative diagnosis, although the clinical course, especially spontaneous improvement without treatment, does not strongly support autoimmune encephalitis. Additionally, the mild inflammatory picture of his CSF on initial presentation in 2017 is suggestive of CAA-ri, as the literature reports that elevated protein and CSF pleocytosis are relatively common among patients with CAA-ri [7,15].

Although a bedside cognitive assessment during his initial presentation to our facility was excluded because of his acute confusional state, a cognitive assessment at his 6-week follow-up might have added value to this case. Finally, as a single case report, generalizability is limited. Despite these constraints, the longitudinal nature of this case provides valuable insight into the potential relapsing-remitting course of CAA-ri.

## Conclusions

CAA-ri is a rare cause of reversible rapidly progressive dementia in elderly patients, often recurring within months but, as in this case, rarely re-emerging after several years. This case underscores the importance of considering CAA-ri in older individuals presenting with rapidly progressive cognitive decline associated with seizures, encephalopathy, focal neurologic deficits, and findings of CMBs and asymmetric vasogenic edema on neuroimaging. Comprehensive neuroimaging is crucial in the timely management of this clinical entity, as it is a noninvasive means of raising clinical suspicion and ensuring early initiation of immunosuppressive therapy, given the practical limitations of obtaining a brain biopsy. Differential diagnoses include autoimmune encephalitis, Creutzfeldt-Jakob disease, and vascular dementia. Careful correlation of clinical features with characteristic MRI findings is essential in distinguishing CAA-ri from these entities and avoiding diagnostic delay.

Prompt initiation of immunosuppressive therapy, particularly high-dose corticosteroids, is essential for the resolution of clinical symptoms and radiologic features and has been shown to reduce the risk of recurrence. In this patient, appropriate treatment resulted in the resolution of clinical symptoms and significant radiologic improvement. This case adds to the existing body of knowledge on CAA-ri and aids in the recognition of this rare entity, which in this case had a relapsing-remitting course with a seven-year interval, and underscores the need for a comprehensive MRI protocol for early identification of this clinical syndrome and for distinguishing it from other causes of reversible rapidly progressive dementia.
